# Extreme miniaturization of a new amniote vertebrate and insights into the evolution of genital size in chameleons

**DOI:** 10.1038/s41598-020-80955-1

**Published:** 2021-01-28

**Authors:** Frank Glaw, Jörn Köhler, Oliver Hawlitschek, Fanomezana M. Ratsoavina, Andolalao Rakotoarison, Mark D. Scherz, Miguel Vences

**Affiliations:** 1 Zoologische Staatssammlung München (ZSM-SNSB), Münchhausenstr. 21, 81247 München, Germany; 2 Hessisches Landesmuseum Darmstadt, Friedensplatz 1, 64283 Darmstadt, Germany; 3 Centrum für Naturkunde, Universität Hamburg, Martin-Luther-King-Platz 3, 20146 Hamburg, Germany; 4 Mention Zoologie et Biodiversité Animale, Université d’Antananarivo, BP 906, 101 Antananarivo, Madagascar; 5 Institute of Biochemistry and Biology, Universität Potsdam, Karl-Liebknecht-Str. 24–25, 14476 Potsdam, Germany; 6 Zoologisches Institut, Technische Universität Braunschweig, Mendelssohnstr. 4, 38106 Braunschweig, Germany

**Keywords:** Evolution, Zoology

## Abstract

Evolutionary reduction of adult body size (miniaturization) has profound consequences for organismal biology and is an important subject of evolutionary research. Based on two individuals we describe a new, extremely miniaturized chameleon, which may be the world’s smallest reptile species. The male holotype of *Brookesia nana* sp. nov. has a snout–vent length of 13.5 mm (total length 21.6 mm) and has large, apparently fully developed hemipenes, making it apparently the smallest mature male amniote ever recorded. The female paratype measures 19.2 mm snout–vent length (total length 28.9 mm) and a micro-CT scan revealed developing eggs in the body cavity, likewise indicating sexual maturity. The new chameleon is only known from a degraded montane rainforest in northern Madagascar and might be threatened by extinction. Molecular phylogenetic analyses place it as sister to *B. karchei*, the largest species in the clade of miniaturized *Brookesia* species, for which we resurrect *Evoluticauda* Angel, 1942 as subgenus name. The genetic divergence of *B. nana* sp. nov. is rather strong (9.9‒14.9% to all other *Evoluticauda* species in the 16S rRNA gene). A comparative study of genital length in Malagasy chameleons revealed a tendency for the smallest chameleons to have the relatively largest hemipenes, which might be a consequence of a reversed sexual size dimorphism with males substantially smaller than females in the smallest species. The miniaturized males may need larger hemipenes to enable a better mechanical fit with female genitals during copulation. Comprehensive studies of female genitalia are needed to test this hypothesis and to better understand the evolution of genitalia in reptiles.

## Introduction

Numerous vertebrate lineages have achieved extremely small body sizes, especially among the ectothermic fish, amphibians, and reptiles. Extremely miniaturized animals are generally thought to face physiological challenges that limit further size reductions^1^. Yet, miniaturization has independently evolved many times. The repeated evolution of such an extreme phenotype suggests that selection can often favour its emergence^1,2^, but currently our understanding of miniaturization and the underlying evolutionary pressures is far from complete. Morphologically, miniaturization is often associated with an evolutionary loss of phalangeal elements, with modifications of the skull and other features like relatively larger eyes and braincases, which often might reflect functional constraints and paedomorphosis^1–5^. To improve the picture, it is essential to complete our basic knowledge of the diversity of diminutive vertebrates.

Two clades of squamate reptiles have independently converged on what seems to be the minimum body size for the order, and indeed for amniotes as a whole^3^: *Sphaerodactylus* dwarf geckos from Central America and *Brookesia* dwarf chameleons from Madagascar. The smallest of these are 14–15 mm in minimum body size (snout–vent length, SVL) of adults^4,5^, but other members of the genera are considerably larger (*S. pacificus* and *B. perarmata* reach maximum male body sizes of 49 mm and 66 mm, respectively^6^). In both genera, the smallest species are characterized by clear paedomorphism, a frequent feature of miniature animals^1^, often arising from heterochrony, and particularly obvious by their relatively large heads and eyes.

The brookesiine chameleon genus *Brookesia* consists of predominantly terrestrial species divided in two major lineages, which diverged from each other ca. 40–50 million years ago^7–9^. One of these lineages includes larger species of 34–66 mm SVL, while the other contains only highly miniaturized species. At present, 12 described species are known from this clade^5,10^, none of which exceeds 30 mm SVL, with the smallest species *B. micra* reaching a maximum adult female SVL of 19.9 mm^5^. A report of live *B. micra* reaching 23 mm SVL^11^ is unfortunately not vouchered and cannot be verified.

Most miniaturized *Brookesia* are rainforest species, which inhabit mostly forests in lowlands (e.g. *B. minima* on Nosy Be) and rarely at higher elevations > 1000 m a.s.l. (e.g. *B. tedi* on Marojejy). Other species prefer dry forest, especially on karstic underground^5,12^. The majority of species exhibit very small ranges, with only few species being known from more than two locations. This microendemism may be related to the complex topography in northern Madagascar where these and other *Brookesia* species are predominantly distributed^13^. Their diminutive size combined with their small ranges have contributed to the fact that much of the diversity of this clade has been overlooked until recently.

Here, we report on the discovery of a new species of *Brookesia* that is apparently still smaller than other miniaturized species of the genus, measuring less than 14 mm SVL in an adult male and 19 mm in a female. We describe this new species and discuss several aspects of miniaturization in these chameleons.

## Results

### Phylogenetic position of the new chameleon species

**Figure 1:**
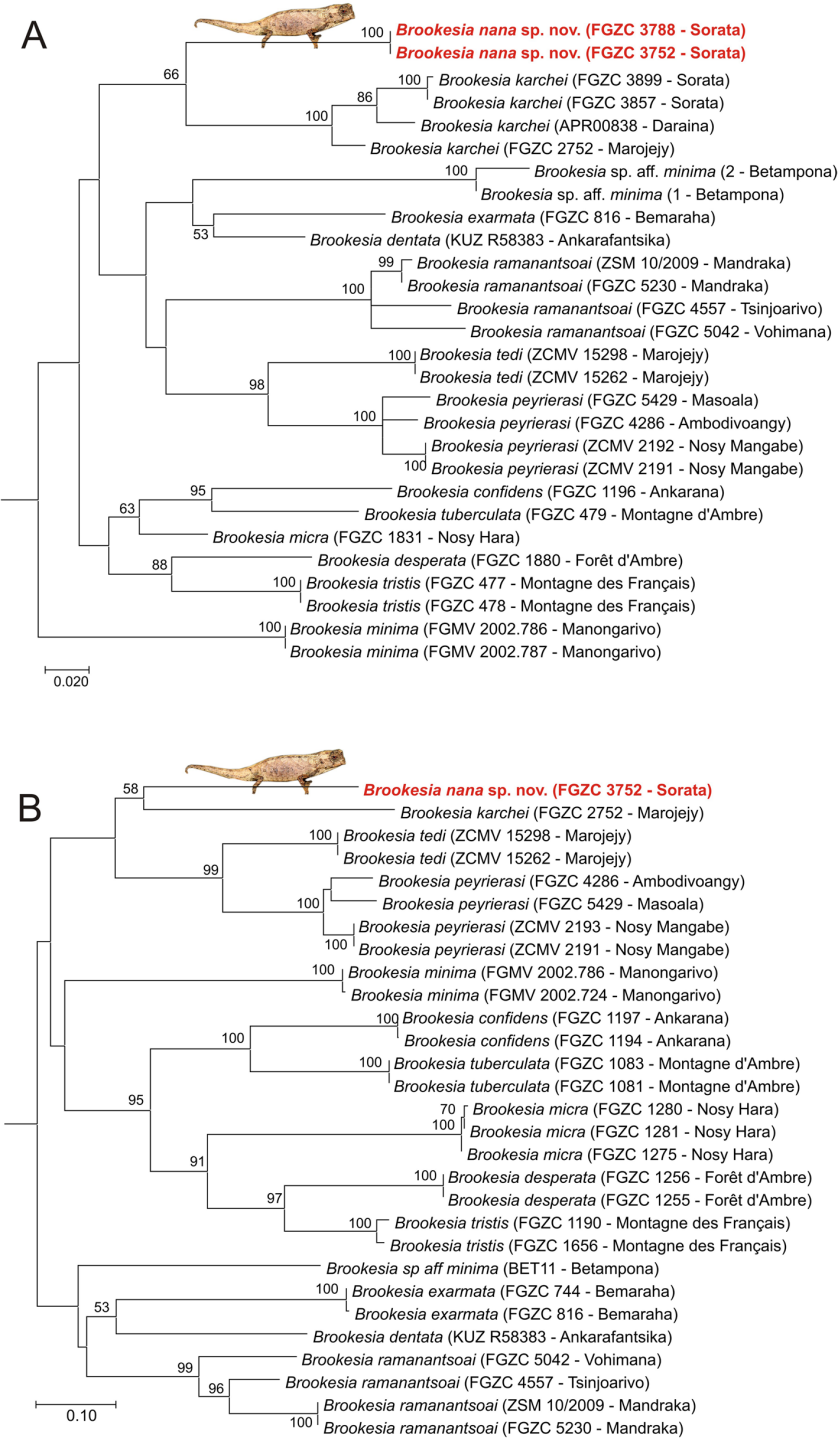
Molecular phylogenetic trees of specimens in the subgenus *Evoluticauda* (known as *Brookesia minima* group), based on sequences of the mitochondrial (**A**) 16S (480 bp) and (**B**) ND2 (571 bp) genes, inferred under the Maximum Likelihood optimality criterion, and the GTR + G (16S) and HKY + I + G (ND2) substitution models. Values at nodes are support values from a bootstrap analysis in percent (500 replicates) and are shown only if > 50%. The two gene fragments were analysed separately and not concatenated because partly different samples were available for each of them. The trees were rooted with *B. brygooi* (removed for better graphical representation).

**Figure 2:**
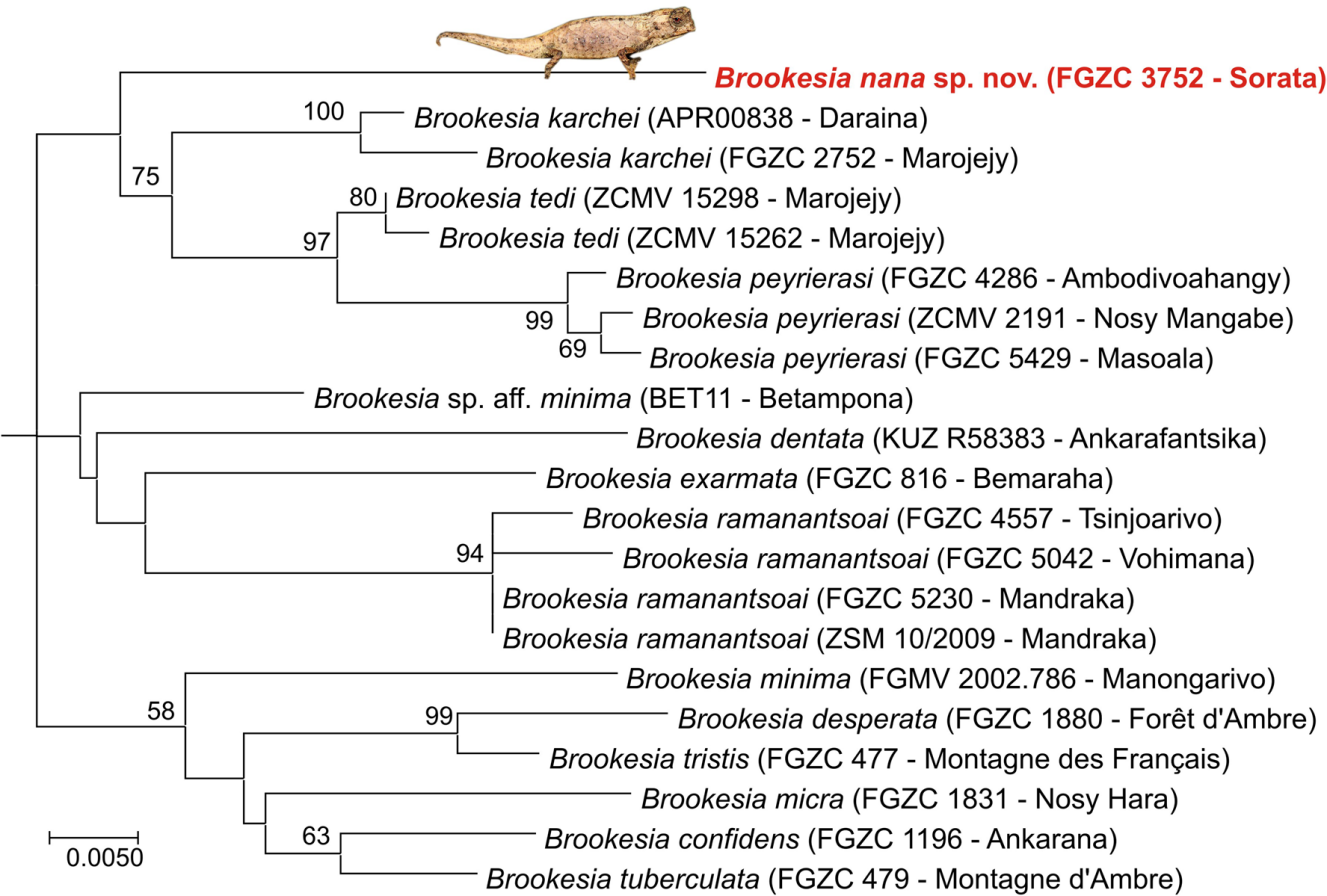
Molecular phylogeny of specimens in the subgenus *Evoluticauda* (known as *Brookesia minima* species group), based on the nuclear CMOS gene (alignment length 847 bp, but only about 400 bp available for all samples) and inferred under the Maximum Likelihood optimality criterion (K2P + G substitution model). Values at nodes are support values from a bootstrap analysis in percent (500 replicates) and are only shown if > 50%. The tree was rooted with *B. brygooi* (removed for better graphical representation).

The Maximum Likelihood (ML) trees obtained from analysis of two mitochondrial gene fragments (16S, ND2: Fig. 1) and one nuclear gene fragment (CMOS: Fig. 2) suggested concordant relationships among species of *Brookesia*, similar to those previously inferred^5,10^, which were based on a more limited taxon sampling. Among the new aspects of our analysis is the confirmation of a specimen from the Masoala Peninsula as *Brookesia peyrierasi.* Also, the new samples of *B. karchei* from Sorata cluster with other samples of this taxon from Marojejy and Daraina, and the new samples of *B. ramanantsoai* from Tsinjoarivo and Vohimana cluster with other samples of this taxon from Mandraka. In all these cases, the samples from the different locations show a substantial genetic divergence: uncorrected pairwise distances (p-distances) in the 16S gene among localities were 3.2‒3.4% for *B. peyrierasi,* 3.4‒4.4% for *B. karchei*, and 4.3‒6.3% for *B. ramanantsoai.*

The two specimens of our focal lineage from Sorata had identical 16S sequences, and in the two mitochondrial trees they were placed sister to *B. karchei* (bootstrap support 58% and 66%), whereas in the CMOS tree they formed the sister group of a clade containing *B. karchei, B. peyrierasi*, and *B. tedi* (bootstrap support 75%). 16S genetic divergences of the Sorata lineage were 9.9‒11.3% to *B. karchei*, and 10.5‒14.9% to all other *B. minima* group species. The Sorata lineage also showed a substantial divergence in the nuclear CMOS gene, which usually is rather conserved among closely related reptiles; uncorrected pairwise distances were 5.1% to *B. karchei,* and 4.0‒7.3% to all other *B. minima* group species.

**Figure 3:**
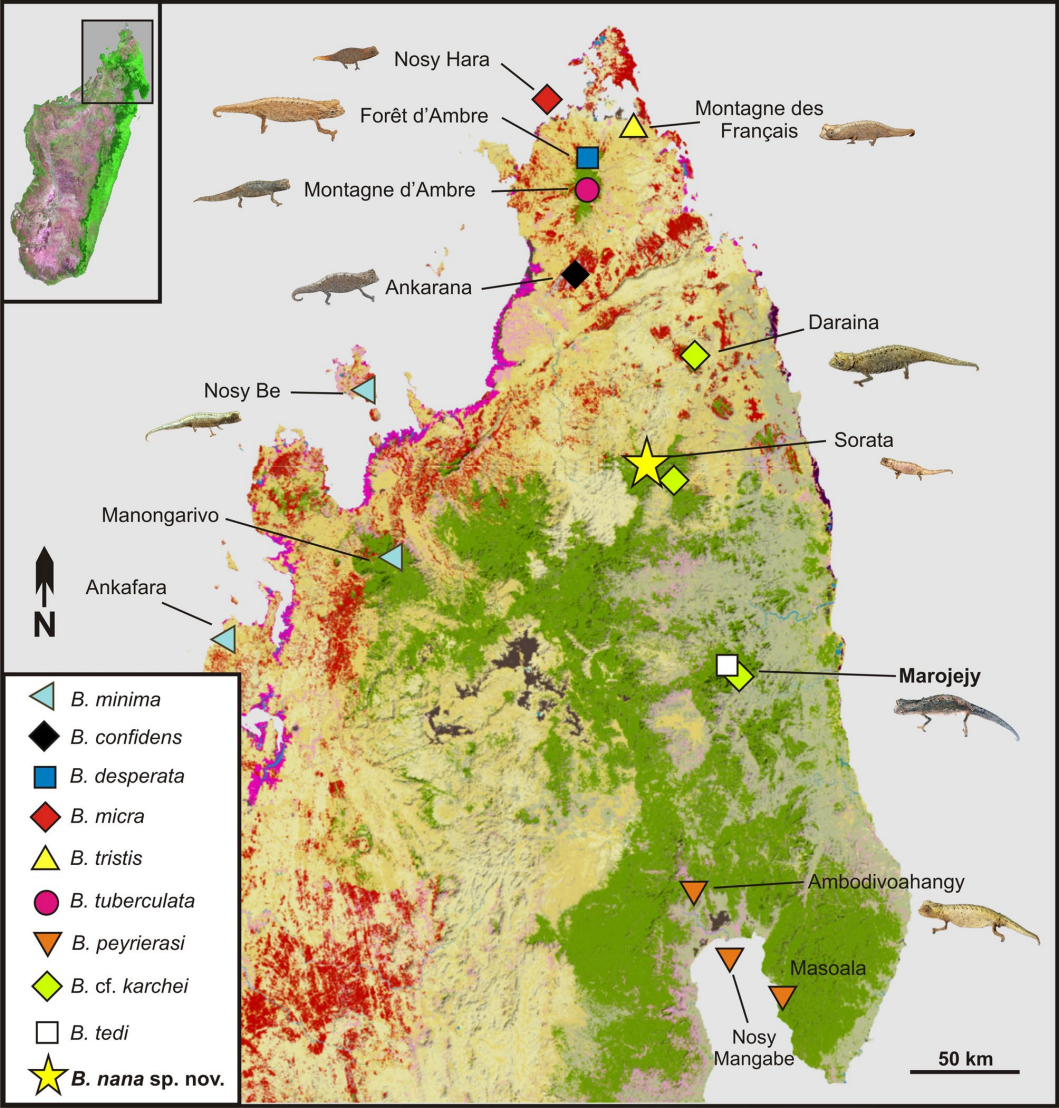
Map of northern Madagascar, showing the distribution of species of the subgenus *Evoluticauda* (known as *Brookesia minima* group) in this region (only showing records verified by molecular data^5,10,14^). Note that *B. dentata, B. exarmata*, and *B. ramanantsoai* occur further south and are not included in the map. Orange (dry forest) and green (rainforest) show remaining primary vegetation in 2003–2006. Modified from the Madagascar Vegetation Mapping Project; http://www.vegmad.org.

A map with genetically confirmed records of the *B. minima* group from northern Madagascar (Fig. 3), based on data from previous publications^5,10,14^, indicates that species are reliably known from a maximum of three localities.

## Systematics

Order Squamata Oppel, 1811^15^.

Family Chamaeleonidae Rafinesque, 1815^16^.

Subfamily Brookesiinae Angel, 1942^17^.

Genus *Brookesia* Gray, 1865^17^.

Subgenus *Evoluticauda* Angel, 1942 (resurrected herein, justification below).

### *Brookesia nana* sp. nov

#### Holotype

ZSM 1660/2012 (field number FGZC 3788), adult male, from near a campsite in the Sorata massif, 13.6851° S, 49.4417° E, ca. 1280 m a.s.l., northern Madagascar, collected on 1 December 2012 by F. Glaw, O. Hawlitschek, T. Rajoafiarison, A. Rakotoarison, F. M. Ratsoavina, and A. Razafimanantsoa.

#### Paratype

UADBA-R/FGZC 3752, adult female, from near the pitfall site 1, Sorata massif, 13.6817° S, 49.4411° E, 1339 m a.s.l., northern Madagascar, collected on 29 November 2012 by same collectors as the holotype.

#### Nomenclatural statement

A Life Science Identifier (LSID) was obtained for the new species (*Brookesia nana*) from ZooBank: urn:lsid:zoobank.org:act: 37B38077-FA5D-48E9-BACF-723061B3921F and for this publication: urn:lsid:zoobank.org:pub: 540F80C8-EC9F-49A9-B13F-77C75DED5962.

### Diagnosis

A diminutive chameleon species assigned to the genus *Brookesia* on the basis of its small body size, short tail, presence of rows of dorsolateral tubercles along vertebral column, presence of pelvic spine, and molecular phylogenetic relationships. *Brookesia nana* sp. nov. is distinguished by the following unique suite of morphological characters: (1) male SVL 13.5 mm, female SVL 19.2 mm; (2) male TL mm 21.6 mm, female TL 28.9 mm; (3) TaL/SVL ratio of 0.51 in male; (4) absence of lateral or dorsal spines on the tail; (5) absence of dorsal pelvic shield in sacral area; (6) presence of distinct pelvic spine; (7) pale brown dorsal colouration with slightly darker markings in life; (8) absence of apical spines on the hemipenis.

Within the genus *Brookesia*, *B. nana* sp. nov. can easily be distinguished from all species that are not members of the *B. minima* species group by its diminutive size (SVL 13.5–19.2 mm vs. > 34 mm). Within the *B. minima* species group, it can be distinguished from most species by the smaller total length (TL). Based on TL (21.6–28.9 mm), both males and females are significantly smaller than all known specimens of *B. desperata* (39.7–47.6 mm), *B. exarmata* (39.8–40.1 mm), *B. karchei* (51.0 mm), and *B. ramanantsoai* (39.0–43.5 mm), and are slightly but distinctly smaller when compared to *B. tristis* (30.7–36.5 mm), *B. confidens* (29.2–36.2 mm), and *B. peyrierasi* (32.2–43.1 mm). Four species of the *B. minima* group are in an overall comparable size range: *B. micra*, *B. minima*, *B. tedi*, and *B. tuberculata*. Yet, the male of the new species (TL 21.6 mm) is the smallest adult *Brookesia* so far known, compared to the previously smallest specimen, a male of *B. micra* with 22.2 mm TL and 15.3 mm SVL.

The very short tail of the male *B. nana* (TaL/SVL 0.51; 0.60 in the female) constitutes a difference to males of most species of the *B. minima* group: male TaL/SVL is 0.60–0.70 in *B. confidens*, 0.59–0.63 in *B. desperata*, 0.66 in *B. karchei*, 0.65–0.73 in *B. minima*, 0.57–0.92 in *B. peyrierasi*, 0.8 in *B. ramanantsoai*, 0.74–0.92 in *B. tedi*, 0.71–0.72 in *B. tristis*, and 0.68–0.88 in *B. tuberculata.*

Given its tiny size, the new species is most similar to *B. micra* (SVL 15.3–15.8 and TL 22.5–23.6 in males; SVL 18.7–19.9 mm and TL 26.9–28.8 in females), which has an even shorter relative tail length in males (TaL/SVL 0.47–0.49). However, males of *B. micra* differ by a more robust habitus; by a flat surface distally forming a symmetrical comb of six large, rounded papillae on the apex of the hemipenis (absent in the new species); and by life colouration, namely a dark brown body and a yellow-orange tail (versus pale brown body and tail with indistinct darker markings). Moreover, molecular data provide evidence for a distant relationship of *B. nana* and *B. micra*.

### Description of the holotype

**Figure 4:**
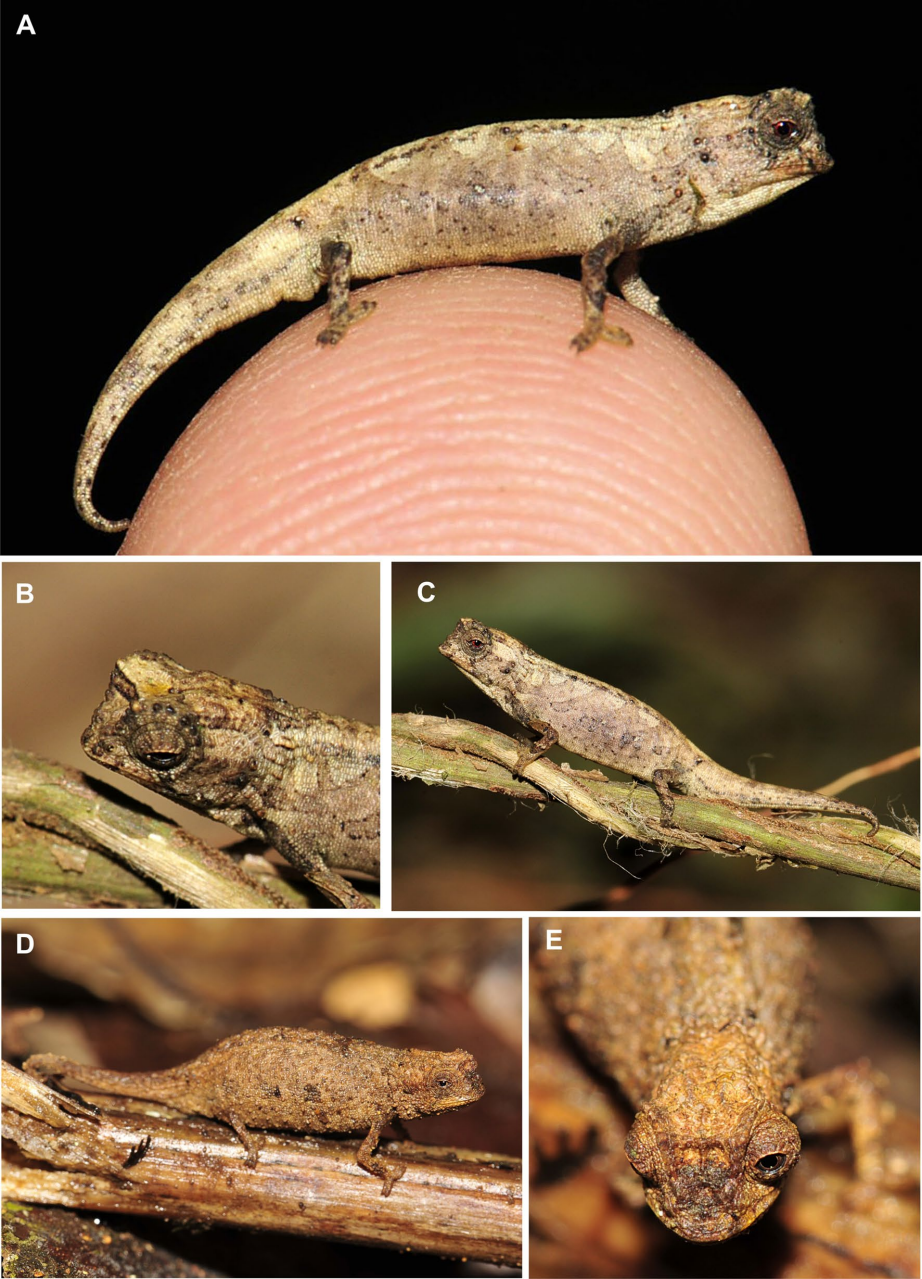
*Brookesia nana* sp. nov. in life. (**A**–**C**) male holotype (ZSM 1660/2012). (**D**, **E**) female paratype (UADBA-R/FGZC 3752).

**Figure 5:**
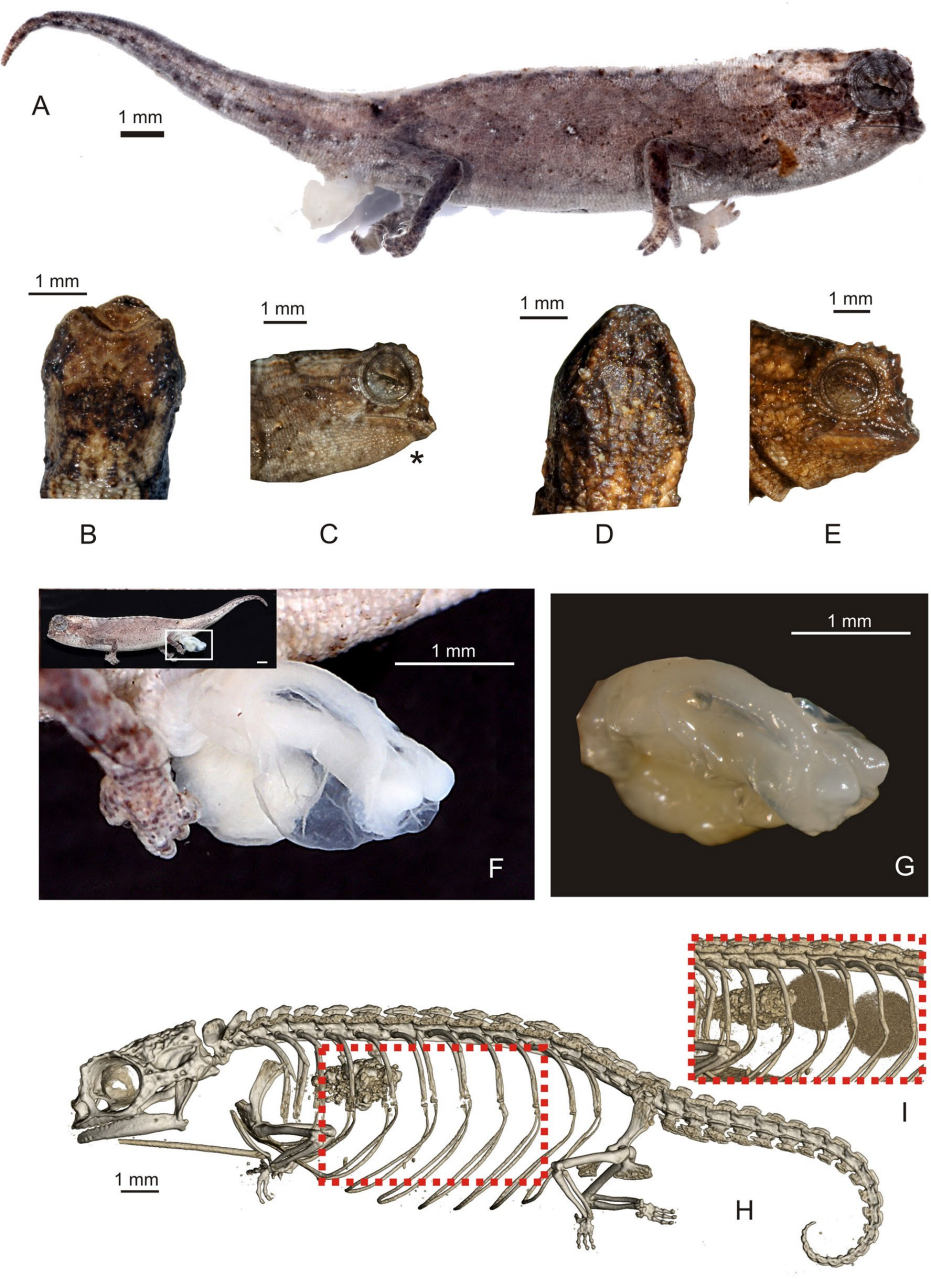
Morphological characters of *Brookesia nana* sp. nov.: (**A**) preserved holotype (ZSM 1660/2012) in lateral view, showing right everted hemipenis, (**B**) head in dorsal and (**C**) lateral (mirrored, indicated with asterisk) views; head of female paratype (UADBA-R/FGZC 3752) in (**D**) dorsal and (**E**) lateral views; (**F**, **G**) close-ups of everted left hemipenis of holotype photographed under different light conditions; (**H**) micro-CT scan image of the female paratype in lateral view showing its skeleton. The inset image (**I**) shows the area marked by the stippled square viewed at a different rendering threshold, showing two developing eggs in the females’ ovaries.

Adult male in excellent state of preservation (Figs. 4A–C, 5). 21.6 mm TL and 13.5 mm SVL. For additional measurements, see Table 1. Lateral crest on head weakly developed, barely recognizable; weak orbital crest; transversal row of enlarged tubercles at the posterior edge of head lacking, no distinct border between head and body, posterior crest lacking; a pair of short curved parasagittal crests that start above the eyes and fade at midlevel of head; depression between the eyes lacking any further crests; one pointed tubercle on each side of head; few scattered, slightly enlarged tubercles on lateral surfaces of head; orbital crest slightly denticulated; distinct supraocular cone absent; supranasal cone very tiny, not projecting beyond tip of snout; head longer than wide; chin and throat without enlarged tubercles. Dorsal surface of body without vertebral ridge or keel; 5/5 (left/right) dorsolateral pointed tubercles that form an incomplete longitudinal line, ending approximately at level of midbody; anteriormost pointed dorsolateral tubercle being largest; pointed dorsolateral tubercles along vertebral column almost equally spaced; dorsal surface of tail lacking distinctly enlarged tubercles; no dorsal pelvic shield in sacral area, but distinct small pelvic spine; lateral surface of body with few irregularly spaced enlarged tubercles; venter without enlarged tubercles; no enlarged pointed tubercles on limbs; no pointed tubercles around cloaca; longitudinal row of slightly enlarged tubercles lateral on anterior tail; no dorsal, lateral, or ventral spines on tail; no enlarged tubercles on ventral surfaces of tail.

**Table 1:** Morphometric measurements of holotype and paratype of *Brookesia nana* sp. nov. (all in mm).

	ZSM 1660/2012	UADBA-R/FGZC 3752
	FGZC 3788	FGZC 3752
	Holotype	Paratype
Sex	M	F
TL	21.6	28.9
SVL	13.5	19.2
TaL	8.1	9.7
HW	2.5	3.0
HH	2.0	2.7
ED	1.3	1.5
FORL	3.9	5.1

See “Materials and Methods” for abbreviations.

Left hemipenis fully everted, right hemipenis almost fully everted (Fig. 5). The fully everted hemipenis is 2.5 mm long, tubular, elongated, with a small flattened apical end with a clear lip around its circumference (Fig. 5F,G). A pair of structures emerge from the apical surface, each of which consists of a fleshy lobe. The truncus is smooth and lacks any trace of calyces.

In life, overall dorsal ground colouration was pale brown, with some lighter blotches in the dorsal and dorsolateral regions, partly extending to the flanks forming incomplete streaks, as well as numerous small dark brown and blackish tubercles and spots. A pattern of four diffuse beige streaks running obliquely from the dorsum to the mid-flanks were recognizable (Fig. 4C). A beige patch was present on the anterior head (Fig. 4B). Two dark streaks ran from the lower margin of the eye to the upper lip. The dorsolateral tubercles and the supraocular crest were blackish. Exterior surfaces of forelimbs and hindlimbs were distinctly darker than flanks and mottled with brown and grey. Darker radial streaks were present on the eyelid, and the iris was dark red (Fig. 4A).

After 6 years in ethanol, the body colouration is generally faded with less evident pattern. The ground colouration is pale brown, becoming distinctly lighter lateroventrally and ventrally. An interrupted dark brown mid-dorsal line runs longitudinally on the dorsum. Head laterally with a diffuse pattern of different shades of brown, grey, and white. Dorsolateral tubercles blackish, pelvic spines whitish. Flanks with dark brown to beige tubercles and patches, including four nearly blackish circles. The dark radial streaks are more distinct than they were in life.

### Variation

Female paratype is in very good state of preservation. Lateral crest on head present, starting at midlevel of eye and stretching backwards to posterior crest; prominent orbital crests; transversal row of enlarged tubercles at the posterior edge of head that separates the head from the body, forming posterior crest; a pair of short curved parasagittal crests that start above the eyes and fade at posterior level of eyes; depression between the eyes with short indistinct median crest and a pair of curved crests starting above eyes and converging to midlevel of head; five pointed tubercles on each side of posterior crest; scattered, slightly enlarged tubercles on lateral surfaces of head; orbital crest denticulated; distinct supraocular cone absent; supranasal cone distinct, small, not projecting beyond tip of snout; head longer than wide; chin and throat without enlarged tubercles. Dorsal surface of body without vertebral ridge or keel; 5/5 (left/right) dorsolateral pointed tubercles along vertebral column, barely recognizable, forming an incomplete longitudinal line; pointed dorsolateral tubercles almost equally spaced; dorsal surface of tail lacking distinctly enlarged tubercles; enlarged tubercles on lateral tail not recognizable; no dorsal pelvic shield in sacral area, but distinct pelvic spine; lateral surface of body with few irregularly spaced enlarged tubercles; venter without enlarged tubercles; scattered enlarged and distinctly pointed tubercles on limbs; no pointed tubercles around cloaca; no dorsal, lateral, or ventral spines on tail; no enlarged tubercles on ventral surfaces of tail. In life, dorsal colour brown, with some darker coloured tubercles, scattered flecks and spots, but generally lacking any conspicuous pattern. Dorsal surface of head slightly paler (Fig. 4D,E). In preservative, the female paratype is generally darker than the holotype, with most enlarged lateral tubercles and numerous small tubercles being dark brown. Dorsal side with a large dark brown patch in its posterior part.

### Etymology

The specific epithet is the Latin noun *nana* (meaning female dwarf) in the nominative singular.

### Conservation status

*Brookesia nana* is known from just two specimens and a single location and thus belongs to the ca. 14% of the world's lizard species that are only known from the type locality^18^. This extremely poor knowledge makes it difficult to reliably evaluate the distribution and the conservation status of this species. However, given that most of the miniaturized *Brookesia* species are microendemic with limited elevational range (Fig. 3), a small range might be also expected for *B. nana*. During our expedition in 2012 the natural habitats of the Sorata massif were highly threatened. At lower elevations, the natural forest had been completely eradicated and anthropogenic pressure at the existing edges was high, especially from deforestation, slash-and-burn agriculture, and cattle. These threats were increasingly extending to higher altitude including the type locality of *B. nana*. Recently, the Sorata massif has received official protection as part of the new protected area ‘Resérve de Ressources Naturelles du Corridor Marojejy-Anjanaharibe Sud-Tsaratanàna partie Nord’, also known as COMATSA Nord^19^. This new reserve may hopefully help to preserve the remaining forest habitats, but the current threat situation around the type locality is unknown. However, according to the current state of knowledge, we suggest that *B. nana* qualifies as Critically Endangered B1ab(iii) under the Red List Criteria of the IUCN^20^ as the extent of occurrence is estimated to be less than 100 km^2^, all individuals occur in one threat-defined location, and there is continuing decline in the extent and quality of its forest habitat in the Sorata massif. We recommend that the extinction risk of this species be assessed officially for the IUCN Red List of Threatened Species as soon as possible. This finding confirms the results of a previous study that Malagasy chameleons have a higher proportion of threatened species compared to other species-rich reptile groups (geckos, skinks, and gerrhosaurids)^21^.

### Genital organ size in Malagasy chameleons

**Figure 6:**
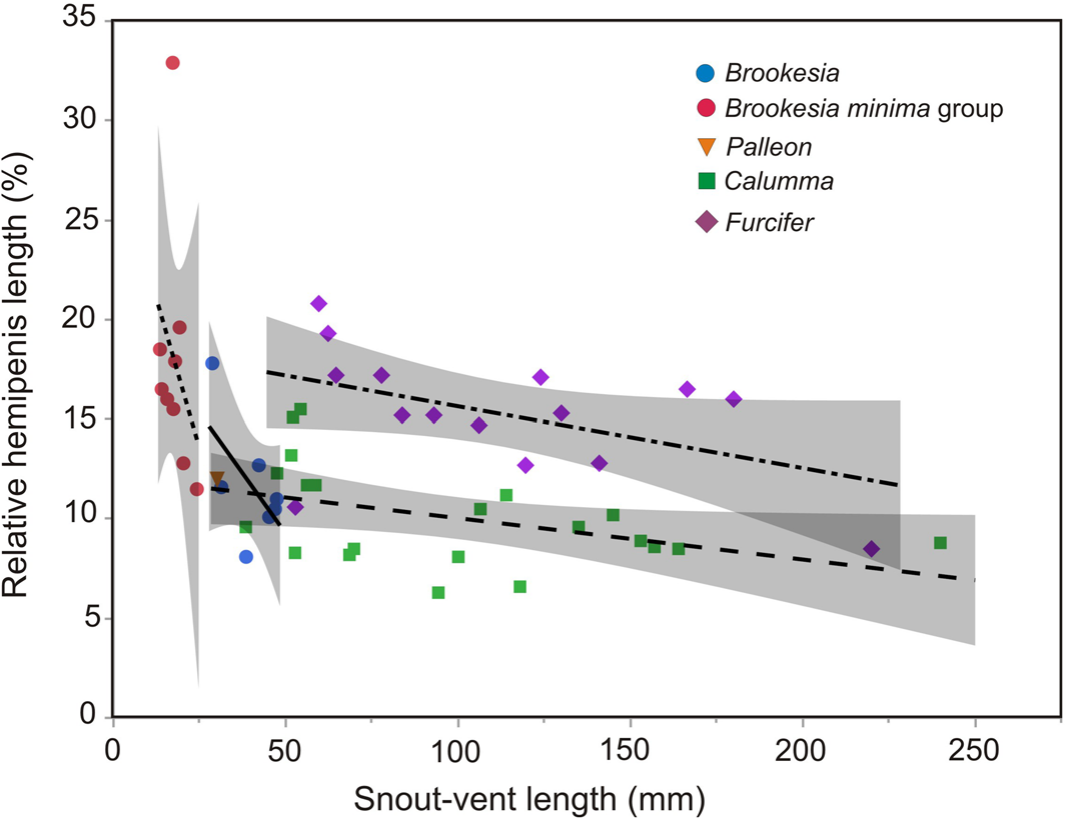
Relation between body size (snout–vent length, SVL) and relative hemipenis length (relative HPL = HPL/SVL in percent), for genera of Malagasy chameleons. The graph shows a prevalent negative correlation of relative HPL with SVL, but also clear differences among genera, especially among the two genera of large-sized tree chameleons, *Calumma* and *Furcifer*. The species with the highest value of relative HPL is *Brookesia tuberculata*.

Relative hemipenial length in Malagasy and Comoran chameleons ranged over more than a half-order of magnitude, from a minimum of 6.3% of SVL in *Calumma capuroni* to 32.9% in *Brookesia tuberculata*, and with an average of 13.1% over the 52 species for which data were available. The value of *B. tuberculata*—with genitals of almost one-third of body length—is, however, exceptional, with the next largest values being around 20% in *B. peyrierasi, Furcifer cephalolepis*, and *F. lateralis*. A non-parametric Spearman rank test confirmed that relative hemipenial length was negatively correlated to snout–vent length (Spearman’s R = − 0.358; P = 0.0085). This overall trend was consistent in all genera, but among the tree chameleons, *Calumma* had distinctly and consistently shorter hemipenes than *Furcifer* (Fig. 6). Although small-sized chameleons had relatively larger hemipenes, the very long genital organs of *B. tuberculata* clearly stood out as an outlier (Fig. 6). The holotype of *Brookesia nana* had the fifth largest relative hemipenial length (18.5%) of the 52 studied species and the third largest in the genus *Brookesia*, supporting that this tiny chameleon is indeed an adult male.

### Resurrection of *Evoluticauda* Angel, 1942 as subgenus of *Brookesia* Gray, 1865

Previous molecular studies have revealed two major lineages in *Brookesia*, which separated from each other ca. 43 million years ago^9^. This split is only slightly younger than the split between the genera *Bradypodion/Nadzikambia*, *Trioceros/Kinyongia* and *Calumma*/*Furcifer* (all ca. 45–46 mya) and older than the split between *Rieppeleon/Archaius* (ca. 35 mya) and between the different subgenera of *Rhampholeon*^9^. Most species of the large-bodied clade of *Brookesia* show very distinct rows of bony lateral projections along the vertebral column, which are based on a unique vertebral structure and might function as body armour to prevent predation^22^. These rows of projections are either absent, incomplete, or very poorly developed in the lineage of small-bodied species, which are also recognizable by their miniaturized adult size (22–51 mm versus 51–110 mm total length) and relatively larger hemipenis length (Fig. 6). Due to the old divergence and the morphological distinctness of the two clades we here suggest to consider them as different subgenera:

Subgenus *Brookesia* Gray, 1865 (large-bodied clade)

Type species: *Chamaeleo superciliaris* Kuhl, 1820.

Contents: *Brookesia antakarana*, *B. bekolosy* (attribution tentative), *B. betschi*, *B. bonsi*, *B. brunoi*, *B. brygooi*, *B. decaryi*, *B. ebenaui*, *B. griveaudi*, *B. lambertoni*, *B. lineata*, *B. perarmata*, *B. stumpffi*, *B. superciliaris*, *B. therezieni*, *B. thieli*, *B. vadoni*, *B. valerieae*.

Distribution: Madagascar.

Subgenus *Evoluticauda* Angel, 1942 (miniaturized clade, known as *B. minima* group)

Type species: *Brookesia tuberculata* Mocquard, 1894.

Contents: *Brookesia confidens*, *B. dentata*, *B. desperata*, *B. exarmata*, *B. karchei*, *B. micra*, *B. minima*, *B. nana*, *B. peyrierasi*, *B. ramanantsoai*, *B. tedi*, *B. tristis*, *B. tuberculata.*

Distribution: Northern half of Madagascar.

## Discussion

### Body size of *Brookesia nana* and *B. micra*

*Brookesia nana* sp. nov. is a remarkable addition to the diversity of microendemic and miniaturized chameleons in northern Madagascar, and with a SVL of only 13.5 mm the holotype represents once more a new record at the lower size limit of amniotes. Until now, the smallest *Brookesia* was *B. micra*, with a confirmed minimal adult male size of 15.3 mm^5^. In a valuable ecological study, Villeneuve^11^ observed 117 *B. micra* and measured the SVL of living individuals to the nearest 1 mm. In this paper, a body size distribution graph was presented in which males and females as small as 9 mm SVL were reported, but these data refer to juveniles, as adults were defined as ≥ 13 mm SVL and juveniles/sub-adults ≤ 13 mm SVL. The largest body size of *B. micra* from this graph was 20 mm for a female, in line with data of Glaw et al.^5^, but two males of 20 and 23 mm represent an important shift of maximal sizes. Unfortunately, these exceptional values are not discussed in this study^11^. We here consider them as in need of confirmation, although we are aware that exceptionally large specimens are known from numerous amphibian and reptile species and may thus be found in *B. micra* and other miniaturized species as well. Villeneuve^11^ also reported on the sexual size dimorphism of *B. micra* and stated in the abstract that he ‘found adult males to have a significantly larger snout‒vent length (SVL) than adult females’, whereas in the results (referring to a table with SVL measurements) it was reported that ‘adult female *B. micra* tended to be larger than adult male individuals’. The latter result is typical for *B. micra* and other species of the subgenus *Evoluticauda*^5^, and the former thus probably an error.

### Evolution and consequences of miniaturized body size

Although all species of miniaturized *Brookesia* belong to the subgenus *Evoluticauda*, important size differences are seen within the group. The mitochondrial trees (Fig. 1) place *B. nana* sister to *B. karchei,* the largest species in *Evoluticauda* (30.7 mm SVL in females^5^). A second extremely small species, *B. micra* (minimum male SVL 15.3 mm, maximum female SVL 19.9 mm) is phylogenetically relatively close to *B. desperata* where females reach 30 mm SVL^5^. The available data do not allow us to unambiguously distinguish in this case between convergent extreme miniaturization in *B. micra* and *B. nana*, or convergent reversal to somewhat larger body sizes in *B. desperata* and *B. karchei*. The obvious presence of homoplasy and/or reversal in the evolution of miniaturization agrees with the observations in other taxa, such as the independent origin of morphologically similar miniaturized taxa in microhylid and other frogs^23–26^, and the evolutionary lability of body size traits in other predominantly small-sized vertebrates^27–29^.

The species of *Brookesia* that apparently have independently evolved their tiny sizes also share a number of other morphological features, such as a general reduction of dorsolateral spines or tubercles along the vertebral column, almost complete lack of head ornaments such as supraocular crests and cones, and short tails. Whether these characteristics are allometric correlates of small body size, e.g. via paedomorphism^30,31^ or may be driven by convergence on a small-size body shape optimum^32^ cannot be decided without a substantial amount of further data and analyses, including a greatly improved knowledge of the morphological variation of both sexes and the ontogenetic development of these characters in juveniles.

Given possible functions of chameleon head ornaments in sexual selection^33,34^ and of the tail in *Brookesia* for assisted walking^35^, it is likely that miniaturization in these lizards is linked to either functional causes, or functional consequences, or both. For example, small chameleon species are known to outperform larger species during ballistic tongue projection^36^, but none of the miniaturized *Brookesia* species has yet been studied in this respect. Also, studies on the microhabitat requirements and ecology of *Brookesia* species are scarce and largely restricted to the larger *Brookesia* species^37,38^, so that the behavioural and ecological consequences of the extreme miniaturization of *Evoluticauda* species remain completely unknown.

Several miniaturized lizards, especially species of *Sphaerodactylus*, occur on islands^4^, as do the smallest species of snakes^39^. It is appealing to relate this to the so-called island rule, much discussed especially for mammals where small mammals tend to evolve larger sizes, and large mammals smaller sizes, compared to their mainland conspecifics^40,41^ (but see ref.^42^). On the contrary, in lizards, it was found that small species on islands become smaller than their mainland conspecifics, while large ones become larger still, opposite to predictions of the island rule^43^. Whether the presence of miniaturized chameleons in Madagascar can be interpreted as supporting this finding is uncertain given that Madagascar, with a surface of about 587,041 km^2^, qualifies more as a microcontinent than an island. Comparing the distribution of the two most strongly miniaturized species, *B. micra* appears to be restricted to the tiny 270 ha islet of Nosy Hara with an estimated population of 100,000 to 150,000 individuals^11^, which may have driven miniaturization. However, the new species *B. nana* occurs in a mountain massif that can be considered rather as part of a major rainforest block of northern Madagascar and its small size is unlikely to be related to specific insularity-related drivers. The elevational distribution of *B. nana* is, however, remarkable in that it is only one of three species in *Evoluticauda* occurring at elevations above 1300 m a.s.l.

### Patterns of fusion of fingers and toes

As all chameleons, *Brookesia* are characterized by a unique pattern of fusion of fingers and toes: on the forelimbs, the outer two and inner three toes are fused, respectively, whereas on the hindlimbs the pattern is reversed. This ‘chamaeleodactyl’ morphology is accompanied by numerous modifications of the mesopodial elements, which however differ among chameleon genera^44^. The small-sized genera of ground chameleons, including the Malagasy *Brookesia* and *Palleon*, but also the African *Rhampholeon* and *Rieppeleon*, were found to maintain the fewest independent carpal and tarsal elements as adults, while the genera of larger-sized arboreal chameleons have a larger number of mesopodial elements, which may be related to locomotor mode^44^. For *Brookesia*, these conclusions were drawn based on an analysis of *B. stumpffi,* a species reaching over 50 mm SVL, while the truly miniaturized ground chameleons have not yet been studied in detail for their hand and foot skeleton. A more comprehensive comparative analysis of skeletal anatomy across *Brookesia* of different sizes may reveal whether differences in hand and foot morphology are related to their splitting from a phylogenetically basal node among chameleons, or by-products of small size and miniaturization, or functionally adaptive in relation to their forest floor habitat.

### Size and evolution of male genitalia

One striking feature of miniaturized chameleons is the relatively large size of their genital organs. This is particularly obvious in the very long hemipenes of *Brookesia tuberculata,* but also the balloon-shaped hemipenes of *B. minima* and *B. ramanantsoai* attain an enormous volume and a width much exceeding body width of these small lizards (photos in ref.^5^). The causes for this allometric relationship are poorly understood. Sexual selection and communication in many chameleons relies on optical signals, both related to colour and external ornaments such as crests, casques, spines, or snout protuberances^33,34,45^. Most Malagasy ground chameleons of the genera *Brookesia* and *Palleon* stand out among other Malagasy chameleons by being small-sized, by their dull colouration and lack of capacity for major colour changes, and their limited amount of external ornamentation. In contrast to the situation in the larger-sized *Calumma* and *Furcifer*, in *Brookesia* the females are typically larger than the males^46^, suggesting that male–male competition may play a more limited role in their mate choice behaviour or that physiological constraints prevent further female size reductions. Across the animal kingdom, extreme sizes of genitals occur. They can be similar to body length in ducks, and up to eight times the body length in barnacles^47^, being usually related to functional necessities (e.g., in sessile barnacles) or sexual conflict and male-male competition in waterfowl^48^. Sexual size dimorphism can strongly influence the evolution of reproductive strategies and can lead to functional conflicts between the sexes, e.g., an evolutionary mismatch between the absolute sizes of male and female genitalia within species, as has been shown for orb-weaving spiders, where genital dimorphism increases with increasing sexual size dimorphism^49^. The distinct differences in relative hemipenis length between large and small chameleon species might be a consequence of the reversal of sexual size dimorphism, given that in larger-sized chameleon genera like *Furcifer* and *Calumma*, males are generally larger than females, whereas the opposite is true in small-sized genera, e.g. *Brookesia* and *Rhampholeon*^46,50^. In these miniaturized species, the smaller males may simply need larger hemipenes to allow for a better mechanical fit that makes successful copulation with the much larger females possible. To test this plausible hypothesis there is an obvious need for comprehensive studies of female genitalia of chameleons and other squamates.

Although the current evidence (M.D. Scherz and collaborators in progress; and data herein) suggests that hemipenial ornamentation in chameleons is predominantly determined by allometric factors related to body size, it is obvious that additional factors play a relevant role causing for instance the distinct differences in hemipenial size among *Calumma* and *Furcifer* species of similar body sizes (Fig. 6). In-depth comparative studies of the mating system of these genera as well as *Brookesia*, and especially of the miniaturized *Brookesia* species with exaggerated genital sizes, emerges as an important priority for future research, in order to fully understand the evolution of these highly specialized lizards, and the evolutionary consequences and drivers of miniaturization in vertebrates.

## Materials and methods

### Fieldwork, permits and morphological measurements

Miniaturized *Brookesia* species were intensively sought during the day on the ground and at night with torchlight. Vouchers were anaesthetised and subsequently euthanised by oral application of lidocaine. This method was carried out in accordance with all relevant guidelines and regulations. No experiments were conducted with the living animals. After taking tissue samples (stored in pure ethanol), vouchers were fixed with 90% ethanol and deposited in 75% ethanol for long-term storage. Collection of specimens was conducted under permit No. 265/12/MEF/SG/DGF/DCB.SAP/SCB (dated 18 Oct. 2012) and exportation of specimens under permit No. 163N-EA12/MG12 (dated 17 Dec. 2012), both issued by the Direction Générale des Forêts (Ministère de l'Environnement, des Eaux et Forêts de la République de Madagascar). Import permits were issued by the German CITES authority (Bundesamt für Naturschutz).

Field numbers (FGZC) refer to the field series of F. Glaw. We deposited the vouchers in the collections of the Mention Zoologie et Biodiversité Animale of the Université d’Antananarivo (UADBA-R) and Zoologische Staatssammlung München (ZSM). Morphometric analysis and morphological descriptions follow a previous study^5^. The following measurements were taken by MV to the nearest 0.1 mm using a digital calliper: TL (total length); SVL (snout–vent length); TAL, tail length; HW, maximum head width; HH, maximum head height; ED, eye diameter; FORL, forelimb length.

The X-ray micro-Computed Tomography (micro-CT) scan was produced using a phoenix|x nanotom m cone beam scanner (GE Measurement & Control, Wunstorf, Germany), with details of the method as described in ref.^51^. Scans were deposited in MorphoSource (https://www.morphosource.org/Detail/ProjectDetail/Show/project_id/953).

### Molecular analysis

For molecular analysis, we used DNA sequences of fragments of the mitochondrial genes for 16S rRNA (16S) and NADH Dehydrogenase Subunit 2 (ND2), and the nuclear gene for oocyte maturation factor mos (CMOS). Our dataset builds upon sequences from a previous study^5^, but with a reduced representation (two sequences per species) for ND2, and also including sequences of one sample of *B. peyrierasi* and two samples of *B. tedi*^10^. This data set was expanded by newly determined sequences of the two available samples of the new species from Sorata, and of several additional samples of *Brookesia karchei, B. peyrierasi,* and *B. ramanantsoai*. Because for some species the individual samples sequenced for ND2 differed from those sequenced for 16S, we refrained from combining these two mitochondrial DNA fragments for analysis. Furthermore, in order to test for genealogical concordance between nuclear and mitochondrial DNA^52^, we also analysed the CMOS sequences separately.

We extracted genomic DNA and amplified the target gene fragments using standard protocols as described previously^5^, with the primers ND2F17 (5′-TGACAAAAAATTGCNCC-3′)^53^ and ALAR2 (5′-AAAATRTCTGRGTTGCATTCAG-3′)^54^ for ND2, 16SA-L (5′-CGCCTGTTTATCAAAAACAT-3′) and 16S-BH (5′-CCGGTCTGAACTCAGATCACGT-3′) for 16S^55^, and CO8 (5′-GCTTGGTGTTCAATAGACTGG-3′) and CO9 (5′-TTGGGAGCATCCAAAGTCTC-3′) for CMOS^56^. We purified PCR products with ExoSAPIT (Thermo Fisher Scientific, Waltham, MA, USA) and sequenced them on an automated DNA sequencer (ABI 3130 XL; Applied Biosystems). We checked, corrected, and trimmed sequences with the software CodonCode Aligner (CodonCode Corporation), and aligned them using the Clustal algorithm in in MEGA7^55^. Newly obtained sequences were submitted to GenBank (accession numbers MK452380‒MK452387, MK45737‒MK457374, and MK457447‒MK457451); for accession numbers of previously published sequences, see refs.^5,10^.

Sequences were analysed in MEGA7^57^. We determined the most suitable substitution models determined under the Bayesian Information Criterion, implemented in MEGA7 (16S: GTR + I + G; ND2: HKY + I + G; CMOS: a K2P + G model), and conducted phylogenetic analyses under the Maximum Likelihood optimality criterion, with nearest-neighbour interchange (NNI) branch-swapping, and with 500 heuristic bootstrap replicates.

### Genital morphology

To understand patterns of allometry in hemipenes of chameleons, we measured body size (snout–vent length) and everted hemipenis length (HPL) in a total of 97 adult males of 52 species of the genera *Brookesia*, *Palleon*, *Calumma* and *Furcifer* from Madagascar and the Comoros (Supplementary Table S1). Where several individuals per species were available, we calculated species averages for both characters for further analysis. Relative hemipenis length, i.e., the ratio between SVL and HPL, was plotted against SVL, and non-parametric correlations calculated with Statistica 7.1 (Statsoft Inc.).

## Supplementary Information

Supplementary Information

## Data Availability

All data generated or analysed during this study are included in this published article (and its Supplementary Information files), MorphoSource, and GenBank.
