# Supplementary material for: Extreme miniaturization of a new amniote vertebrate and insights into the evolution of genital size in chameleons
Source: Sci Rep. 2021 Jan 28;11:2522. doi: 10.1038/s41598-020-80955-1 (PMC7844282; doi:10.1038/s41598-020-80955-1)
Supplement: Supplementary file 1 — Supplementary Information [file 41598_2020_80955_MOESM1_ESM.docx]

**Extreme miniaturization of a new amniote vertebrate and insights into the evolution of genital size in chameleons**

Frank Glaw, Jörn Köhler, Oliver Hawlitschek, Fanomezana M. Ratsoavina, Andolalao Rakotoarison, Mark D. Scherz & Miguel Vences

**Supplementary Table S1**. Snout-vent length (SVL), hemipenial length (HPL) and relative hemipenial length in percent (HPL/SVL*100) for 97 adult male specimens of 52 chameleon species from Madagascar and the Comoros.

| **Species** | **Voucher specimen** | **SVL (mm)** | **HPL (mm)** | **Relative**  **HPL (%)** |
| --- | --- | --- | --- | --- |
| *Brookesia antakarana* | ZSM 225/2004 | 45.5 | 3.7 | 8.1 |
| *Brookesia antakarana* | ZSM 234/2004 | 51.4 | 4.5 | 8.8 |
| *Brookesia antakarana* | ZSM 226/2004 | 42.4 | 4.4 | 10.4 |
| *Brookesia antakarana* | ZSM 1661/2012 | 47.3 | 4.8 | 10.1 |
| *Brookesia antakarana* | ZSM 1664/2012 | 48.2 | 5.6 | 11.6 |
| *Brookesia antakarana* | ZSM 153/2018 | 45.6 | 4.7 | 10.3 |
| *Brookesia antakarana* | ZSM 103/2003 | 44.3 | 4.4 | 9.9 |
| *Brookesia antakarana* | ZSM 1652/2012 | 51.3 | 7.1 | 13.8 |
| *Brookesia antakarana* | ZSM 1669/2012 | 50.2 | 7.5 | 14.9 |
| *Brookesia betschi* | ZSM 430/2005 | 28.7 | 5.1 | 17.8 |
| *Brookesia brunoi* | ZSM 888/2010 | 38.5 | 3.1 | 8.1 |
| *Brookesia decaryi* | ZSM 560/2001 | 46.6 | 4.5 | 9.7 |
| *Brookesia decaryi* | ZSM 558/2001 | 47.1 | 5.3 | 11.3 |
| *Brookesia desperata* | ZSM 2170/2007 | 24.6 | 3.1 | 12.6 |
| *Brookesia desperata* | ZSM 2171/2007 | 22.8 | 2.3 | 10.1 |
| *Brookesia desperata* | ZSM 2176/2007 | 23.7 | 2.7 | 11.4 |
| *Brookesia desperata* | ZSM 2177/2007 | 25.7 | 3.0 | 11.7 |
| *Brookesia micra* | ZSM 1507/2008 | 13.7 | 2.1 | 15.3 |
| *Brookesia micra* | ZSM 2183/2007 | 14.2 | 2.5 | 17.6 |
| *Brookesia minima* | ZSM 1709/2004 | 16.7 | 2.9 | 17.4 |
| *Brookesia minima* | ZSM 930/2003 | 19.0 | 3.5 | 18.4 |
| *Brookesia nana* | ZSM 1660/2012 | 13.5 | 2.5 | 18.5 |
| *Brookesia peyrierasi* | ZSM 447/2010 | 20.0 | 3.0 | 15.0 |
| *Brookesia peyrierasi* | ZSM 436/2005 | 20.0 | 4.1 | 20.5 |
| *Brookesia peyrierasi* | ZSM 437/2005 | 17.7 | 4.2 | 23.7 |
| *Brookesia peyrierasi* | ZSM 435/2005 | 19.0 | 3.7 | 19.5 |
| *Brookesia ramanantsoai* | ZSM 10/2009 | 20.3 | 2.6 | 12.8 |
| *Brookesia stumpffi* | ZSM 1681/2012 | 42.6 | 4.6 | 10.8 |
| *Brookesia stumpffi* | ZSM 500/2014 | 42.4 | 4.6 | 10.8 |
| *Brookesia stumpffi* | ZSM 562/2001 | 41.6 | 6.9 | 16.6 |
| *Brookesia superciliaris* | ZSM 316/2000 | 45.8 | 4.5 | 9.8 |
| *Brookesia superciliaris* | ZSM 448/2010 | 43.9 | 4.3 | 9.8 |
| *Brookesia superciliaris* | ZSM 377/2016 | 46.0 | 4.9 | 10.7 |
| *Brookesia tedi* | ZSM 438/2016 | 15.6 | 2.5 | 16.0 |
| *Brookesia tristis* | ZSM 1505/2008 | 17.4 | 2.7 | 15.5 |
| *Brookesia tuberculata* | ZSM 499/2000 | 16.7 | 6.2 | 37.1 |
| *Brookesia tuberculata* | ZSM 1708/2004 | 18.0 | 5.5 | 30.6 |
| *Brookesia tuberculata* | ZSM 1039/2003 | 17.0 | 5.3 | 31.2 |
| *Brookesia vadoni* | ZSM 440/2016 | 32.2 | 3.0 | 9.3 |
| *Brookesia vadoni* | ZSM 621/2009 | 29.7 | 4.2 | 14.1 |
| *Brookesia vadoni* | ZSM 124/2005 | 32.0 | 3.7 | 11.6 |
| *Calumma amber* | ZSM 165/2008 | 103.4 | 10.1 | 9.8 |
| *Calumma amber* | ZSM 40/2003 | 109.5 | 12.3 | 11.2 |
| *Calumma ambreense* | ZSM 1042/2003 | 157.0 | 13.5 | 8.6 |
| *Calumma brevicorne* | ZSM 1043/2003 | 145.0 | 14.8 | 10.2 |
| *Calumma capuroni* | ZSM 132/2005 | 94.3 | 5.9 | 6.3 |
| *Calumma cucullatum* | ZSM 655/2014 | 164.0 | 14.0 | 8.5 |
| *Calumma gallus* | ZSM 321/2000 | 44.6 | 6.2 | 13.9 |
| *Calumma gallus* | ZSM 456/2010 | 55.6 | 5.9 | 10.6 |
| *Calumma gallus* | ZSM 622/2009 | 42.2 | 5.4 | 12.8 |
| *Calumma gastrotaenia* | ZSM 506/2014 | 55.5 | 7.7 | 13.9 |
| *Calumma gastrotaenia* | ZSM 510/2014 | 61.3 | 5.5 | 9.0 |
| *Calumma gastrotaenia* | ZSM 459/2010 | 58.9 | 7.3 | 12.4 |
| *Calumma gehringi* | ZSM 43/2016 | 50.9 | 7.7 | 15.1 |
| *Calumma gehringi* | ZSM 2843/2010 | 53.2 | 8.0 | 15.0 |
| *Calumma gehringi* | ZSM 1835/2010 | 51.6 | 6.8 | 13.2 |
| *Calumma glawi* | ZSM 2042/2008 | 56.2 | 6.6 | 11.7 |
| *Calumma globifer* | ZSM 141/2016 | 135.0 | 12.9 | 9.6 |
| *Calumma guillaumeti* | ZSM 442/2016 | 54.3 | 8.4 | 15.5 |
| *Calumma hafahafa* | ZSM 617/2009 | 118.0 | 7.8 | 6.6 |
| *Calumma linotum* | ZSM 1683/2012 | 52.7 | 4.4 | 8.3 |
| *Calumma malthe* | ZSM 71/2005 | 114.0 | 12.8 | 11.2 |
| *Calumma oshaughnessyi* | ZSM 683/2003 | 152.0 | 15.9 | 10.5 |
| *Calumma oshaughnessyi* | ZSM 1048/2003 | 154.0 | 11.3 | 7.3 |
| *Calumma parsonii* | ZSM 151/2016 | 240.0 | 21.2 | 8.8 |
| *Calumma peltierorum* | ZSM 518/2014 | 103.0 | 8.3 | 8.1 |
| *Calumma peltierorum* | ZSM 1725/2010 | 97.2 | 7.9 | 8.1 |
| *Calumma roaloko* | ZSM 244/2018 | 38.4 | 3.7 | 9.6 |
| *Calumma tarzan* | ZSM 219/2010 | 69.8 | 5.9 | 8.5 |
| *Calumma vencesi* | ZSM 50/2011 | 68.5 | 5.6 | 8.2 |
| *Furcifer angeli* | ZSM 219/2016 | 139.0 | 21.0 | 15.1 |
| *Furcifer angeli* | ZSM 220/2018 | 121.0 | 18.9 | 15.6 |
| *Furcifer antimena* | ZSM 625/2000 | 106.1 | 15.6 | 14.7 |
| *Furcifer balteatus* | ZSM 795/2003 | 166.0 | 21.4 | 12.9 |
| *Furcifer balteatus* | ZSM 682/2003 | 167.0 | 33.7 | 20.2 |
| *Furcifer campani* | ZSM 316/2006 | 61.9 | 5.4 | 8.7 |
| *Furcifer campani* | ZSM 72/2005 | 43.6 | 5.8 | 13.3 |
| *Furcifer cephalolepis* | ZSM 701/2000 | 62.3 | 12.0 | 19.3 |
| *Furcifer lateralis* | ZSM 185/2004 | 61.4 | 9.4 | 15.3 |
| *Furcifer lateralis* | ZSM 543/2001 | 60.3 | 14.9 | 24.7 |
| *Furcifer lateralis* | ZSM 785/2003 | 57.0 | 12.9 | 22.6 |
| *Furcifer major* | ZSM 175/2004 | 83.8 | 12.7 | 15.2 |
| *Furcifer nicosiai* | ZSM 84/2006 | 119.6 | 15.2 | 12.7 |
| *Furcifer oustaleti* | ZSM 665/2014 | 220.0 | 18.8 | 8.5 |
| *Furcifer pardalis* | ZSM 177/2018 | 141.0 | 18.1 | 12.8 |
| *Furcifer petteri* | ZSM 1706/2012 | 84.4 | 13.5 | 16.0 |
| *Furcifer petteri* | ZSM 2181/2007 | 73.1 | 13.2 | 18.1 |
| *Furcifer petteri* | ZSM 2022/2008 | 73.3 | 15.3 | 20.9 |
| *Furcifer petteri* | ZSM 1002/2003 | 80.2 | 11.5 | 14.3 |
| *Furcifer timoni* | ZSM 179/2018 | 96.0 | 14.8 | 15.4 |
| *Furcifer timoni* | ZSM 180/2018 | 90.0 | 13.5 | 15.0 |
| *Furcifer verrucosus* | ZSM 959/2003 | 180.0 | 28.8 | 16.0 |
| *Furcifer voeltzkowi* | ZSM 223/2018 | 124.0 | 21.2 | 17.1 |
| *Furcifer willsii* | ZSM 455/2016 | 61.3 | 12.3 | 20.1 |
| *Furcifer willsii* | ZSM 446/2005 | 72.2 | 12.2 | 16.9 |
| *Furcifer willsii* | ZSM 261/2016 | 60.2 | 8.8 | 14.6 |
| *Palleon nasus* | ZSM 84/2004 | 30.1 | 3.6 | 12.0 |
